# Literary Property—Dr. Gooch and Dr. Burrows

**Published:** 1830-01-01

**Authors:** 


### MISCELLANIES.

### LII.

### Literary Property.

*Dr. Gooch and Dr. Burrows.*

Suum cuique.

When the article (VI.) on Puerperal Mania, in our last number, (p 359—370) was actually passing through the press, and while the proof-sheet was undergoing correction, we received a letter from Dr. Burrows, complaining that several passages had been taken by Dr. Gooch from his work on insanity almost verbatim, and without any literary acknowledgment. Dr. Burrows pointed out several of these parallelisms, or rather plagiarisms, and one of the most striking of these will be found appended to our review, just at the end, page 370. It certainly did appear to us that such parallelisms could hardly be accidental, and we saw no reason why the literary spoliation, as it then appeared to be, should not be pointed out. We had not time to make any other inquiry than merely a comparison of the passages, and it never once occurred to us that Dr. Burrows, who so strenuously claimed property surreptitiously taken from him, was actually reclaiming that which he himself had taken, without acknowledgment, from the accused author! Yet such was the case. On our return to town, after an absence of some months, we found letters from Dr. Gooch, pointing out the identical passage claimed by Dr. Burrows, published ten years previously in his paper on Puerperal Insanity, in the Transactions of the College of Physicians. We immediately showed the parallelisms to Dr. Burrows, who handsomely and candidly acknowledged that he was entirely in the wrong, and that a circumstance pointed out in his work (the loss of his notes) was the cause of his attributing a plagiarism to Dr. Gooch, which Dr. Gooch never committed—but which, in fact, Dr. Gooch might fairly retort on Dr. Burrows.

For our own parts, we have no reason to accuse ourselves of acting improperly either in word or intention; and indeed Dr. Gooch has not reproached us with any thing of the kind. The parallelisms appeared too close to be accidental—and so they turned out. We trust that this will be a lesson to all writers, and more especially compilers, not to be so very positive about their literary property, when their memories are not sufficiently retentive of that which actually belongs to them.

